# Proteomic profile response of *Paracoccidioides lutzii* to the antifungal argentilactone

**DOI:** 10.3389/fmicb.2015.00616

**Published:** 2015-06-18

**Authors:** Renata S. Prado, Alexandre M. Bailão, Lívia C. Silva, Cecília M. A. de Oliveira, Monique F. Marques, Luciano P. Silva, Elisângela P. Silveira-Lacerda, Aliny P. Lima, Célia M. Soares, Maristela Pereira

**Affiliations:** ^1^Laboratório de Biologia Molecular, Instituto de Ciências Biológicas, Universidade Federal de GoiásGoiânia, Brazil; ^2^Laboratório de Produtos Naturais, Instituto de Química, Universidade Federal de GoiásGoiânia, Brazil; ^3^Laboratório de Espectrometria de Massa (PBI), Centro Nacional de Pesquisa de Recursos Genéticos e Biotecnologia, Empresa Brasileira de Pesquisa AgropecuáriaBrasília, Brazil; ^4^Laboratório de Genética Molecular e Citogenética Humana, Instituto de Ciências Biológicas, Universidade Federal de GoiásGoiânia, Brazil

**Keywords:** *Paracoccidioides lutzzi*, paracoccidioidomycosis, proteomic, argentilactone, antifungal

## Abstract

The dimorphic fungi *Paracoccidioides* spp. are the etiological agents of paracoccidioidomycosis (PCM), a mycosis of high incidence in Brazil. The toxicity of drug treatment and the emergence of resistant organisms have led to research for new candidates for drugs. In this study, we demonstrate that the natural product argentilactone was not cytotoxic or genotoxic to MRC5 cells at the IC_50_ concentration to the fungus. We also verified the proteomic profile of *Paracoccidioides lutzii* after incubation with argentilactone using a label free quantitative proteome nanoUPLC-MS^E^. The results of this study indicated that the fungus has a global metabolic adaptation in the presence of argentilactone. Enzymes of important pathways, such as glycolysis, the Krebs cycle and the glyoxylate cycle, were repressed, which drove the metabolism to the methylcytrate cycle and beta-oxidation. Proteins involved in cell rescue, defense and stress response were induced. In this study, alternative metabolic pathways adopted by the fungi were elucidated, helping to elucidate the course of action of the compound studied.

## Introduction

The fungi of the genus *Paracoccidioides* are thermally dimorphic and cause paracoccidioidomycosis (PCM), a human systemic mycosis prevalent in residents of Latin America (Brummer et al., 1993). In Brazil, systemic mycoses are a major cause of mortality considering infectious diseases and the PCM contributes by more than half of the deaths caused by fungal infections (Prado et al., 2009). An essential step for the establishment of the *Paracoccidioides* spp. infection is the transition from mycelium to the yeast form. The fungus lives in the environment as mycelial form, which produces propagules that can be inhaled by the host where change to the yeast phase, causing the infection (Franco, 1987).

Due to toxicity of drug treatment (Travassos et al., 2008) and the appearance of resistance strains (Hahn et al., 2003), new therapeutic approaches for the treatment of PCM have been suggested (Rittner et al., 2012). Natural compounds, synthetic, and semi-synthetic derivatives with antifungal activity against *Paracoccidioides* spp. have been investigated (Johann et al., 2012; Zambuzzi-Carvalho et al., 2013). Argentilactone, the major component of *Hyptis ovalifolia* essential oil, a natural Brazilian plant, inhibits the growth of *P. lutzii* yeast cells, the dimorphism, and the activity of the glyoxylate cycle key enzyme isocitrate lyase (*Pb*ICL) (Prado et al., 2014). In addition, argentilactone inhibits the proliferation of *Cryptococcus neoformans*, *Candida albicans*, *Tricophyton rubrum*, *Tricophyton mentagrophyte, Microsporum gypseum*, and *Microsporum canis* (Oliveira et al., 2004).

Several antifungals drugs act by mechanisms poorly understood. New approaches such as genomics and proteomics were used to investigate the mode of action of new antifungal agents (Mercer et al., 2011; Chan et al., 2012), to identify new targets (Bruneau et al., 2003; Kley, 2004; Hooshdaran et al., 2005; Delom et al., 2006; Rogers et al., 2006; Hoehamer et al., 2010), and to study the synergistic effects among compounds (Xu et al., 2009; Agarwal et al., 2012). This approach was also used to investigate the clinical action of antifungals and new drugs against *Paracoccidioides* spp. (Zambuzzi-Carvalho et al., 2013; Neto et al., 2014).

The study aimed to investigate the cytotoxicity and genotoxicity of argentilactone, as well as, the proteomic profile of *P. lutzii* after incubation with argentilactone. In addition, the work aimed to evaluate the lipids and glucose levels, and *in vivo* methylcitrate dehydrogenase transcript level in *P. lutzii*.

## Experimental

### Extraction of (R)-argentilactone (2H-pyran-2-one, 6-(1-heptenyl)-5,6-dihydro-,[r-(z)])

The essential oil of *H. ovalifolia* was obtained as described previously and the NMR data are consistent with the literature (Oliveira et al., 2004).

### Reduction of 3-(4,5- dimethylthiazol-2-yl)-2,5-diphenyl tetrazolium bromide (MTT) method

The MTT colorimetric method described by Mosmann (1983) was used to evaluation of the cell viability after treatment with 9, 18, 36, and 72 μg/mL argentilactone. The cell viability was measured by the mitochondrial dehydrogenase enzyme activity of living cells. Human lung fibroblast normal cell line (MRC5; CCL-171) used in this study were obtained from the American Type Culture Collection—ATCC, Rockville, Maryland. For the MTT assay, 1 × 10^4^ cells were seeded in 96 well microtiter plates in the absence or presence of argentilactone and incubated at 37°C at atmospheric pressure containing 5% CO_2_. After incubation for 24 h, 10 μL MTT (5 mg/mL) was added to the cells, and following 4 h of incubation with MTT, 200 μL PBS/20% SDS (sodium dodecyl sulfate) was added. A quantification of optical density was measured using a spectrophotometer (Awareness Technology, Palm City, Florida). The percentage of cell viability was calculated by GraphPad Prism 4.02 software (GraphPad Software, San Diego, California).

### Comet assay

The effect genotoxic of argentilactone was examined by comet assay according to Singh et al. (1988). Argentilactone was added at concentrations of 9, 18, 36, and 72 μg/mL to 1 × 10^5^ MRC5 cells and was incubated at 37°C for 24 h. After incubation, 15 μL of the cells was added to 100 μL of a low melting point agarose (0.5%), spread onto microscope glass slides pre-coated with a normal melting point agarose (1.5%), and covered with a coverslip. The slides were incubated for 15 min at 4°C and after were immersed in cold lysis solution (2.4 M NaCl; 100 mM EDTA; 10 mM Tris, 10% dimethylsulfoxide, and 1% Triton-X, pH 10) for 24 h. After lysis, the slides were subjected to electrophoresis for 25 min at 25 V and 300 mA. Thereafter, the slides were neutralized for 15 min in buffer 0.4 M Tris–HCl, pH 7.5, dried at room temperature and fixed in 100% ethanol for 5 min. The slides were stained using 20 μg/mL ethidium bromide. Two slides were prepared for MRC5, and 50 cells were screened per sample using a fluorescence microscope interfaced with a computer. Analysis of the nucleoids was performed in software Comet Score 15 according to the migration of the fragments, as previously described (Kobayashi et al., 1995). The damage index was calculated according to Tice et al. (2000).

### *P. lutzii* and culture conditions

*P. lutzii* (ATCC-MYA-826) has been extensively studied in different laboratories (Pereira et al., 2010; Cruz et al., 2011; Oliveira et al., 2013; Teixeira et al., 2013). The fungus was cultivated in Fava-Netto's medium (1.0% w/v peptone, 0.5% w/v yeast extract, 0.3% w/v proteose peptone, 0.5% w/v beef extract, 0.5% w/v NaCl, 4% w/v glucose, and 1.4% w/v agar, pH 7.2) (Fava-Netto and Raphael, 1961) at 36°C for growth of the yeast phase.

### Culture and cell viability

*P. lutzii* yeast cells were sub-cultured for 1 week in solid Fava-Netto's medium at 36°C. For viability experiments, yeast cells were cultured in a liquid chemically defined medium McVeigh Morton (MMcM) (Restrepo and Jiménez, 1980) in the absence or presence of a sub-inhibitory concentration of 9 μg/mL argentilactone (Prado et al., 2014) at 36°C. Aliquots were collected after 0, 6, 8, 10, and 12 h of incubation. The cell viability was determined by counting stained cells in a Neubauer chamber using trypan blue, based on the principle that live cells with intact cellular membranes expelled the dye (Strober, 2001). All experiments were performed in triplicate.

### Preparation of protein extracts

*P. lutzii* yeast cells were collected after 10 h of contact with 9 μg/mL argentilactone and the total proteins were extracted. Centrifugation of the cells was performed at 10,000 g for 15 min at 4°C and disrupted by glass beads. The extraction buffer (20 mM Tris- HCl pH 8.8; 2 mM CaCl_2_) added of a mixture of protease inhibitors (serine, cysteine and calpain inhibitors) (GE Healthcare, Uppsala, Sweden) was added to the yeast cells. After the addition of glass beads (0.45 mm), the cells were vigorously mixed for 1 h at 4°C, followed by centrifugation at 10,000 g for 15 min at the same temperature. The supernatant was collected, and the protein concentrations were determined by the Bradford reagent (Sigma Aldrich, St. Louis, Missouri). The samples were stored in aliquots at 80°C.

### Protein digestion and label free quantitative nanoUPLC-MS^E^proteomics

Equimolar amount of three biological replicates were pooled were pooled and submitted to the proteomic analysis. A total of 300 μg of each sample in 50 mM ammonium bicarbonate was submitted to tryptic digestion. First, 25 μL of the surfactant RapiGEST™ (0.2% v/v) (Waters Corp, Milford, Massachusetts) was added and then incubated at 80°C for 15 min. The protein samples were reduced with 2.5 μL of a 100 mM DTT solution for 30 min at 60°C; and then alkylated with 2.5 μL of 300 mM iodoacetamide in the dark for 30 min. After, 10 μL of 50 ng/μL (in 50 mM ammonium bicarbonate) trypsin solution (Promega, Madison, Wisconsin) was added. The sample was digested at 37°C overnight. Following the digestion, RapiGEST™ was hydrolyzed with 10 μL of 5% (v/v) trifluoroacetic acid at 37°C for 90 min. The sample was centrifuged at 10,000 g at 4°C for 30 min, and the supernatant was transferred to a Total Recovery vial (Waters Corp). The digests were dried and the peptides were resuspended in 20 mM ammonium formate pH 10. The obtained peptides were further separated by RP-RP-HPLC using a nanoACQUITY™ system (Waters Corp), as described before (Geromanos et al., 2009). Each sample was run in three technical replicates. The column loads were 5 μg of protein digests for the analysis of samples in triplicate. First, the samples were separated in 5 fractions in the mobile phase at pH 10. Each fraction was further separated by reverse phase chromatography with a mobile phase at pH 2.5. Label-free data-independent scanning (MS^E^) experiments were performed with a Synapt HDMS mass spectrometer (Waters, Manchester, UK), which switched between low collision energy MS (3 eV) and elevated collision energies MS^E^ (12–40 eV) applied to the trap “T-wave” CID cell with argon gas (Curty et al., 2014).

The protein identifications and quantitative packaging were generated using specific algorithms (Silva et al., 2005, 2006) and search was performed against a *P. lutzii* specific database. The ProteinLynx Global server v.2.5.2 (PLGS) with Expression^E^ informatics v.2.5.2 was used to proper spectral processing, database searching conditions and quantitative comparisons. The database was randomized to access the false-positive rate of identification (4%). Trypsin was the primary digest reagent, allowing for 1 missed cleavage. Carbamidomethyl-C was specified as fixed modification and phosphorylation STY and oxidation M were used as variable modifications. The minimum fragment ion matches per peptide, the minimum fragment ion matches per protein, and the minimum peptide matches per protein were, respectively set as 2.5 and 1. It was used 50 ppm as mass variation tolerance. A protein detected in all replicates presenting a variance coefficient less than 10% was used to normalize the expression data to compare the protein levels between control and argentilactone-treated conditions. The confidence interval of 95% was used. The mass spectrometry proteomics data have been deposited to the ProteomeXchange Consortium (Vizcaíno et al., 2014) via the PRIDE partner repository with the dataset identifier PXD002285.

### Cell culture and macrophage infection assay

The J774 A1 macrophage cells were cultured in 75 m^2^ flasks and incubated at 37°C with 5% CO_2_ in an RPMI medium (RPMI 1640, Vitrocell, São Paulo, São Paulo) supplemented with 10% (v/v) fetal bovine serum. The J774 macrophage cells were plated at 5 × 10^5^ cells per well on 6-well culture plates and infected with *P. lutzii* yeast cells at a 1:5 ratio macrophage:yeast. The cells were co-cultivated for 12 h at 37°C in 5% CO_2_ to allow for fungi adhesion and/or internalization. After this, the treatment with 9 μg/mL argentilactone and controls in the absence of argentilactone and presence of sulfametoxazole were conducted.

### RNA extraction, cDNA synthesis, and quantitative real time reverse transcription PCR (qRT-PCR) analysis

The samples of *P. lutzii* infected macrophages in the presence of 9 μg/mL argentilactone and 0.01 mg/mL sulfametoxazole (control) were washed three times with sterile water. After centrifugation, the pellets were frozen in liquid nitrogen. The cells were disrupted with glass beads for 10 min in the presence of Trizol reagent (Invitrogen™, Carlsbad, California) according to the manufacturer's instructions. The cDNAs were obtained using Superscript II reverse transcriptase (Invitrogen) and an oligo (dT)_15_primer. The qRT-PCR reactions were performed in triplicates of three independent experiments using a StepOnePlus™ RT-PCR system (Applied Biosystems, Foster City, California). The SYBR green PCR master mix (Applied Biosystems) was used as the reaction mixture, with 10 pmol of each primer and 40 ng of template cDNA at a final volume of 25 μL. A melting curve analysis and electrophoresis were performed to confirm a single PCR product. The qRT-PCR thermal cycling consisted of 40 cycles of 95°C for 15 s and 60°C for 1 min. Constitutively expressed alpha tubulin (sense: GAGCGATTCATTGGAGGGATT; anti-sense: ATCAGGGAAAACAGAGTAAGTC) (Zambuzzi-Carvalho et al., 2013) was selected to normalize the samples. A non-template control was included to eliminate contamination or non-specific reactions. The standard curve was generated from a pool of cDNA from each sample. The standard cDNA was serially diluted in a ratio of 1:5. The relative expression levels of selected genes were calculated using the standard curve method for relative quantification (Bookout et al., 2006). The oligonucleotides used in the qRT-PCR analyses are relatives to the methylcitrate dehydrogenase gene (sense: CAACTCTGACCTTGCATTTGAT; anti-sense: GATGTTGAAAGCACCGTTGAC). The experiments were performed in triplicate. A Student's *t*-test was performed to analyze significant differences between the different samples and a *p*-value *p* < 0.05 was considered as significant.

### Dosage of glucose

The concentration of glucose was determined following the instructions of the enzymatic glucose kit (Doles Ltda, Goiânia, Goiás). A total of 1 × 10^5^ cells was treated with 9 μg/mL of argentilactone by 0, 2, 4, 6, 8, 10, 12 and 24 h. The control cells were grown in the absence of argentilactone. Aliquots of 10 μL were collected in each time, adding 1 mL of color solution and incubated for 5 min at 37°C. The absorbance was measured by spectrophotometer at 510 nm.

### Determination of intracellular lipid content

Intracellular lipid content was determined by flow cytometry using lipophilic dye Nile Red. Aliquots were collected after 0, 6, 10, 12 and 24 h of incubation with 9 μg/mL of argentilactone and in the absence of the compound. The cells were washed twice with PBS and incubated with 2 μg/mL Nile red (Sigma Aldrich), for 15 min at room temperature. Nile red intracellular fluorescence was determined by guava easyCyte™ Flow Cytometers (Merck Millipore, Billerica, EUA) on emission channel of 585 nm and excitation 488 nm. A total of 5000 cells were collected to analysis.

## Results and discussion

### Evaluation of argentilactone cytotoxicity against human cells

**Figure 1:**
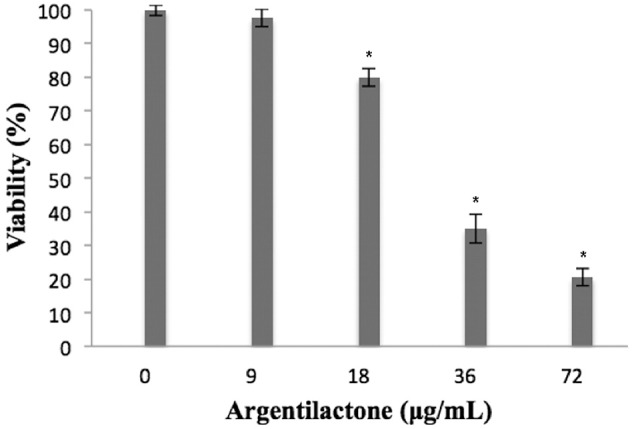
**Percentage of viable MRC5 normal human cells after exposure to different concentrations of argentilactone**. Significance was accepted ^*^p < 0.05. Analysis was performed by a One-Way ANOVA followed by a Tukey post-test.

The cytoxicity of argentilactone was evaluated for human cells MRC5 (Figure 1). The data show a dose-dependent relationship between the number of dead cells and argentilactone concentration. The concentration of 9 μg/mL argentilactone did not promote cell cytotoxicity for MRC5. For the MRC5 cells, the IC_50_ was 32 μg/mL. For the *P. lutzii* yeast cells, the IC_50_ was 18 μg/mL (Prado et al., 2014). These data suggest that the argentilactone is more toxic to the fungus than for human cells.

**Figure 2:**
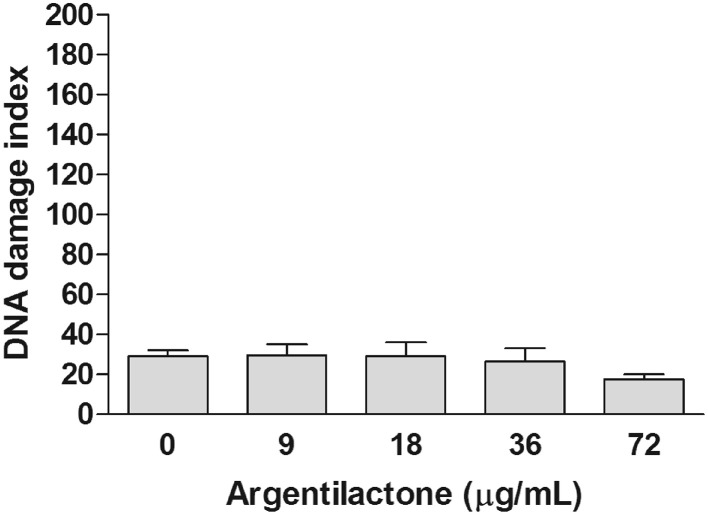
**Effect of argentilactone on the induction of MRC5 cells DNA damage**. Cells were treated with 9, 18, 36, and 72 μg argentilactone for 24 h and analyzed by comet assay. Analysis was performed by a One-Way ANOVA followed by a Tukey post-test.

Aiming to evaluate if argentilactone induces DNA damage in human cells, the comet assay was performed to MRC5 cells treated with different concentrations of this compound. This assay has achieved the status of a standard test in the battery of tests used to assess the safety of novel pharmaceuticals or other chemicals and is now well-established as a sensitive assay for detecting strand breaks in the DNA of single cells (Fairbairn et al., 1995). Figure 2 shows the effect of argentilactone in MRC5 cells. In the MRC5 normal cells the compound did not induce DNA damage when compared to the negative control (*p* > 0.05). The data above suggest that this compound is safe to human.

### Determination of incubation time with argentilactone

Metabolic response and survival strategies of *P. lutzii* were discussed at the molecular level using genomic and proteomic approaches (Desjardins et al., 2011; Weber et al., 2012; Grossklaus et al., 2013; Zambuzzi-Carvalho et al., 2013). In this study, we investigated the response of *P. lutzii* to the antifungal prototype argentilactone.

**Figure 3:**
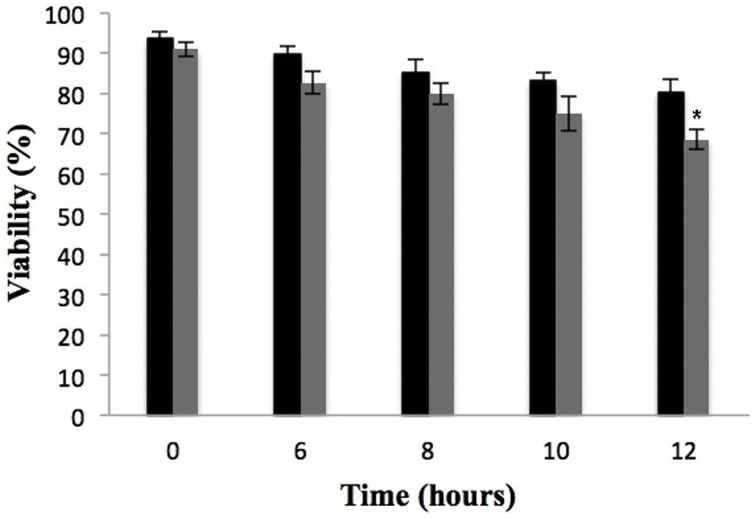
**Effect of argentilactone on** ***P. lutzii*** **cells growth**. Yeast cells were cultured at 36°C in the absence (black) and presence (gray) of 9 μg/mL argentilactone for 12 h. Aliquots were taken and the cells were counted in a Neubauer chamber. ^*^p < 0.05.

A viability curve of *P. lutzii* yeast cells was constructed at time 0, 6, 8, 10, and 12 h in the presence of a sub-inhibitory concentration of 9 μg/mL argentilactone aiming to determine the time point to be used for the proteomic experiments. The time of 10 h with a cell viability of 90% (Figure 3) was chosen for proteomic studies.

### Proteomic response of *P. lutzii* upon exposure to argentilactone

A nanoUPLC-MS^E^-based proteomics approach was employed to identify the *P. lutzii* yeast cell differentially regulated proteins in response to argentilactone. A total of 211 proteins were identified of which 155 had significant regulation at a 1.2-fold change or more. This cut off ratio was used in order to identify broader cellular processes regulated by the compound instead to focus in specifically regulated proteins. From these, 32 were more abundant, 88 less abundant, 20 detected only in treated cells and 15 detected only in the control. A total of 7% of the proteins had no predicted function; the other 93% were classified in functional categories using the FunCat2 system. The regulated proteins were clustered in proteins with increased expression after incubation with argentilactone (Table 1) and proteins with decreased expression after incubation with argentilactone (Table 2).

**Table 1:** ***P. lutzii*** **more abundant proteins after incubation with argentilactone**.

**Functional category^a^**	**Protein description**	**Accession number^b^**	**Protein score**	**Fold change**
**METABOLISM**				
Amino acid metabolism	1-pyrroline-5-carboxylate dehydrogenase	PAAG_05253	1803.48	1.768
	4-aminobutyrate aminotransferase	PAAG_00468	1503.69	1.804
	Homogentisate 1,2-dioxygenase	PAAG_08164	739.59	1.878
	Methylmalonate-semialdehyde dehydrogenase	PAAG_07036	1184.68	1.336
	*O*-acetylhomoserine (Thiol)-lyase	PAAG_08100	3986.80	1.323
	Serine hydroxymethyltransferase	PAAG_08512	1347.52	1.221
	Pyruvate decarboxylase	PAAG_02050	1361.75	1.323
	Sulfite oxidase	PAAG_07811	1481.90	1.221
	Aminopeptidase B	PAAG_09004	450.66	^*^
	Aspartyl aminopeptidase	PAAG_04205	526.39	^*^
	Aspartyl aminopeptidase	PAAG_00664	568.78	^*^
	Cysteine synthase	PAAG_07813	412.94	^*^
	Hydroxymethylglutaryl-CoA lyase	PAAG_06215	1087.40	^*^
	Formate dehydrogenase-III	PAAG_03599	1012.38	^*^
Carbohydrate metabolism	Triosephosphate isomerase	PAAG_02585	10825.00	1.246
	Pyruvate dehydrogenase complex component Pdx1	PAAG_00666	997.13	1.768
	Pyruvate dehydrogenase complex	PAAG_00050	877.32	1.616
	Fumarate reductase Osm1	PAAG_04851	2013.07	1.234
	4-hydroxyphenylpyruvate dioxygenase	PAAG_07875	4971.33	1.568
	N-acetylglucosamine-phosphate mutase	PAAG_01931	398.11	^*^
	Aldehyde dehydrogenase	PAAG_05392	399.42	^*^
	Fumarylacetoacetase	PAAG_08163	2404.04	1.234
Nitrogen metabolism	Formamidase	PAAG_03333	1620.07	1.209
Nucleotide metabolism	Rad4 family protein	PAAG_05019	2058.26	1.377
Coenzyme metabolism	Riboflavin synthase subunit alpha	PAAG_01934	554.30	^*^
Cell rescue, defense and virulence	Proteasome component C5	PAAG_00866	1414.16	^*^
	Superoxide dismutase [Cu-Zn]	PAAG_04164	2348.64	1.297
	Sulfur metabolite repression control protein C	PAAG_07339	4835.50	^*^
**ENERGY**				
Eletron transport	Cytochrome c oxidase polypeptide VI	PAAG_07246	2376.00	1.477
	Cytochrome c oxidase polypeptide IV	PAAG_06796	711.90	
Associate energy conservation	Cytochrome c PriAC=F2TJX0	PAAG_06268	1307.21	1.522
Glycolysis and gluconeogenesis	6-phosphogluconolactonase	PAAG_05621	688.50	1.297
Glyoxylate cycle	Malate synthase	PAAG_04542	617.40	^*^
Krebs cycle	Succinyl-CoA:	PAAG_05093	770.38	^*^
Methyl citrate cycle	2-methylcitrate dehydratase	PAAG_04559	20407.15	1.297
Oxidation of fatty acids	Enoyl-CoA hydratase	PAAG_06309	3244.11	1.716
	Acetyl-CoA acetyltransferase	PAAG_03447	1578.04	^*^
	Peroxisomal 3-ketoacyl-coA thiolase	PAAG_03689	1248.76	^*^
Siderophore-iron transport	Siderophore peptide synthase	PAAG_02354	1582.68	^*^
Protein fate	Chaperone DnaK	PAAG_01339	14261.80	1.259
	Chaperonin	PAAG_05142	71219.03	1.584
	Chaperonin GroL	PAAG_08059	36257.80	1.336
	GrpE protein homolog	PAAG_06255	6685.85	1.649
	Glutathione S-transferase	PAAG_08162	766.51	1.405
	Peptidylprolyl isomerase	PAAG_05788	3381.68	1.284
	CORD and CS domain-containing protein	PAAG_02973	1899.14	^*^
Miscellaneous	Thiol methyltransferase	PAAG_06955	1027.93	1.391
Translation	Endoribonuclease L-PSP	PAAG_08313	12115.91	1.234
Unclassified	Uncharacterized protein	PAAG_00297	870.53	1.649
	Uncharacterized protein	PAAG_07772	1786.94	1.209

a *Functional category—based on the MIPS Functional categories database and GO*.

b *Accession number—accession number of matched protein from Paracoccidioides database (http://www.broadinstitute.org/annotation/genome/paracoccidioides_brasiliensis/MultiHome.html)*.

* *Proteins detected only during incubation with argentilactone*.

**Table 2:** ***P. lutzii*** **less abundant proteins after incubation with argentilactone**.

**Functional category^a^**	**Protein description**	**Accession number^b^**	**Protein score**	**Fold change**
**METABOLISM**				
Amino acid metabolism	Acetolactate synthase	PAAG_00221	849.43	0.726
	Argininosuccinate synthase	PAAG_07114	6934.38	0.522
	Cobalamin-independent methionine synthase MetH/D	PAAG_07626	2518.10	0.577
	Isovaleryl-CoA dehydrogenase, mitochondrial	PAAG_04102	953.65	0.811
	NADP-specific glutamate dehydrogenase	PAAG_07689	1723.70	0.600
	Ornithine aminotransferase	PAAG_06431	1262.54	0.684
	Lysine decarboxylase-like protein	PAAG_03537	800.11	^*^
	NAD-specific glutamate dehydrogenase	PAAG_01002	1969.33	^*^
	Saccharopine dehydrogenase	PAAG_02693	1249.65	^*^
	Serine hydroxymethyltransferase	PAAG_07412	4659.48	0.677
Carbohydrate metabolism	Mannitol-1-phosphate dehydrogenase	PAAG_06473	4920.79	0.726
	Eukaryotic phosphomannomutase	PAAG_00889	1400.74	0.691
	GDP-mannose pyrophosphorylase A	PAAG_08174	860.91	^*^
	Transketolase TktA	PAAG_04444	2581.21	0.763
Coenzyme metabolism	Adenosylhomocysteinase	PAAG_02859	14585.17	0.440
	Dihydropteroate synthase	PAAG_01324	870.83	0.779
	Pyridoxine biosynthesis protein pyroA	PAAG_07321	2354.97	0.787
	S-adenosylmethionine synthase	PAAG_02901	6069.65	0.357
Nucleotide metabolism	Bifunctional purine biosynthesis protein ADE17	PAAG_00731	4517.89	0.811
	Adenylosuccinate lyase	PAAG_04974	686.76	^*^
	S-methyl-5-thioadenosine phosphorylase	PAAG_01302	1274.52	^*^
	UDP-N-acetylglucosamine pyrophosphorylase	PAAG_06885	768.34	0.779
Phosphate metabolism	Inorganic pyrophosphatase	PAAG_00657	4020.00	0.771
Cell cycle and dna processing	Cell division cycle protein 48	PAAG_05518	1782.70	0.719
	D-tyrosyl-tRNA(Tyr) deacylase	PAAG_03334	22078.91	0.741
	Nascent polypeptide-associated complex subunit alpha	PAAG_04571	4281.41	0.779
	Peptidyl-prolyl cis-trans isomerase	PAAG_06168	2417.21	0.795
	Proliferating cell nuclear antigen	PAAG_00923	5676.48	0.748
	TCTP family protein	PAAG_09083	23693.70	0.463
	Thioredoxin	PAAG_02364	25560.74	0.719
	UV excision repair protein Rad23	PAAG_04949	1953.93	0.651
Cell rescue, defense and virulence	Heat shock protein 30	PAAG_00871	6591.33	0.492
	Heat shock protein 88	PAAG_07750	15855.80	0.811
	Heat shock protein SSB	PAAG_07775	5550.62	0.487
**ENERGY**				
Eletron transport and membran associate energy conservation	ATP synthase D chain, mitochondrial	PAAG_04570	1983.65	0.748
	ATP synthase gamma chain	PAAG_05576	4554.87	0.595
	ATP synthase subunit alpha	PAAG_04820	17850.35	0.670
	ATP synthase subunit beta	PAAG_08037	19311.82	0.726
Glycolysis and gluconeogenesis	Phosphoenolpyruvate carboxykinase AcuF	PAAG_08203	2953.75	0.554
	Pyruvate dehydrogenase E1 component alpha subunit	PAAG_08295	904.41	0.748
	Glucokinase glkA	PAAG_06172	746.76	^*^
	Phosphoglucomutase	PAAG_02011	2057.00	0.482
	Phosphoglycerate kinase	PAAG_02869	3428.25	0.619
	Pyruvate kinase	PAAG_06380	9829.55	0.657
	Enolase	PAAG_00771	39472.25	0.779
	Phosphofructokinase subunit	PAAG_01583	587.46	^*^
	Pyruvate dehydrogenase E1 component beta subunit	PAAG_01534	2794.88	0.733
Glyoxylate cycle	Isocitrate lyase	PAAG_04549	923.71	0.827
Krebs cycle	Malate dehydrogenase	PAAG_00053	47991.24	0.795
	Malate dehydrogenase	PAAG_08449	7490.87	0.756
	Isocitrate dehydrogenase subunit 1	PAAG_00856	1820.37	^*^
	Isocitrate dehydrogenase subunit 2	PAAG_07729	1604.29	^*^
	Succinate dehydrogenase flavoprotein subunit, mitochondrial	PAAG_01725	1798.19	0.827
Oxidation of fatty acids	Short-chain specific acyl-CoA dehydrogenase	PAAG_05454	1028.15	^*^
Transport	Carbonic anhydrase	PAAG_05716	854.25	0.795
	Clathrin light chain	PAAG_08252	1049.51	0.741
	GTP-binding nuclear protein ran-1	PAAG_04651	3676.19	0.527
	Nipsnap family protein	PAAG_05960	4593.91	0.677
	Vesicular-fusion protein sec17	PAAG_06233	559.70	^*^
	Rab GDP-dissociation inhibitor	PAAG_06344	1958.40	0.625
Protein fate	G-protein comlpex beta subunit CpcB	PAAG_06996	2600.70	0.741
	Protein disulfide-isomerase	PAAG_00986	14896.18	0.670
Miscellaneous	Thiol-specific antioxidant	PAAG_03216	4271.92	0.427
Translation	Cytosolic large ribosomal subunit protein L30	PAAG_01050	6746.86	0.756
	40S ribosomal protein S0	PAAG_02111	10467.63	0.741
	40S ribosomal protein S11	PAAG_06367	3129.84	0.512
	40S ribosomal protein S14	PAAG_01433	1642.34	0.712
	40s ribosomal protein s15	PAAG_04690	6547.01	0.625
	40s ribosomal protein s26	PAAG_07847	9205.88	0.477
	40S ribosomal protein S5	PAAG_05484	5524.47	0.625
	40S ribosomal protein S7	PAAG_07182	7212.75	0.670
	40S ribosomal protein S8	PAAG_00264	3915.07	0.651
	40S ribosomal protein S9	PAAG_01435	2407.07	0.487
	40S ribosomal protein S9	PAAG_03828	2402.26	0.502
	60S ribosomal protein L13	PAAG_06320	5338.78	0.589
	60S ribosomal protein L15	PAAG_00969	4623.56	0.468
	60S ribosomal protein L18A	PAAG_00952	3245.52	0.571
	60S ribosomal protein L2	PAAG_00430	2292.83	0.517
	60S ribosomal protein L43	PAAG_06569	12650.10	0.543
	60S ribosomal protein L4-A	PAAG_08888	5405.80	0.619
	60S ribosomal protein L5	PAAG_00548	911.54	0.795
	60S ribosomal protein L7	PAAG_06487	3961.73	0.748
	60S ribosomal protein	PAAG_01834	4195.17	0.538
	60S ribosomal protein L31E	PAAG_04965	2514.61	^*^
	Ribosomal protein S23	PAAG_00385	923.83	^*^
	Elongation factor 1-alpha	PAAG_02024	13081.64	0.284
	Elongation factor 1-beta	PAAG_03028	26825.63	0.427
	Elongation factor 1-gamma	PAAG_03556	9096.78	0.317
	Elongation factor 2	PAAG_00594	11304.17	0.403
	Polyadenylate-binding protein	PAAG_00244	1647.87	0.631
	Ribosomal protein L19	PAAG_08497	3909.55	0.607
	Ribosomal protein P0	PAAG_00801	2669.74	0.560
	Ribosomal protein S20	PAAG_03322	1872.13	0.763
	Ribosomal protein S6	PAAG_02634	1918.03	0.589
	40S Ribosomal protein S3	PAAG_01785	6921.45	0.595
	U5 small nuclear ribonucleoprotein component	PAAG_07785	372.29	0.242
Unclassified	Hypothetical protein	PAAG_07955	2234.10	0.507
	Uncharacterized protein	PAAG_07989	907.76	0.657
	Uncharacterized protein	PAAG_04274	841.04	0.726
	Uncharacterized protein	PAAG_02434	1488.40	^*^
	Uncharacterized protein	PAAG_07841	11750.10	0.458
	Uncharacterized protein	PAAG_00724	3895.92	0.492

a *Functional category—based on the MIPS Functional categories database and GO*.

b *Accession number—accession number of matched protein from Paracoccidioides database (http://www.broadinstitute.org/annotation/genome/paracoccidioides_brasiliensis/MultiHome.html)*.

* *Proteins detected only in control conditions*.

**Figure 4:**
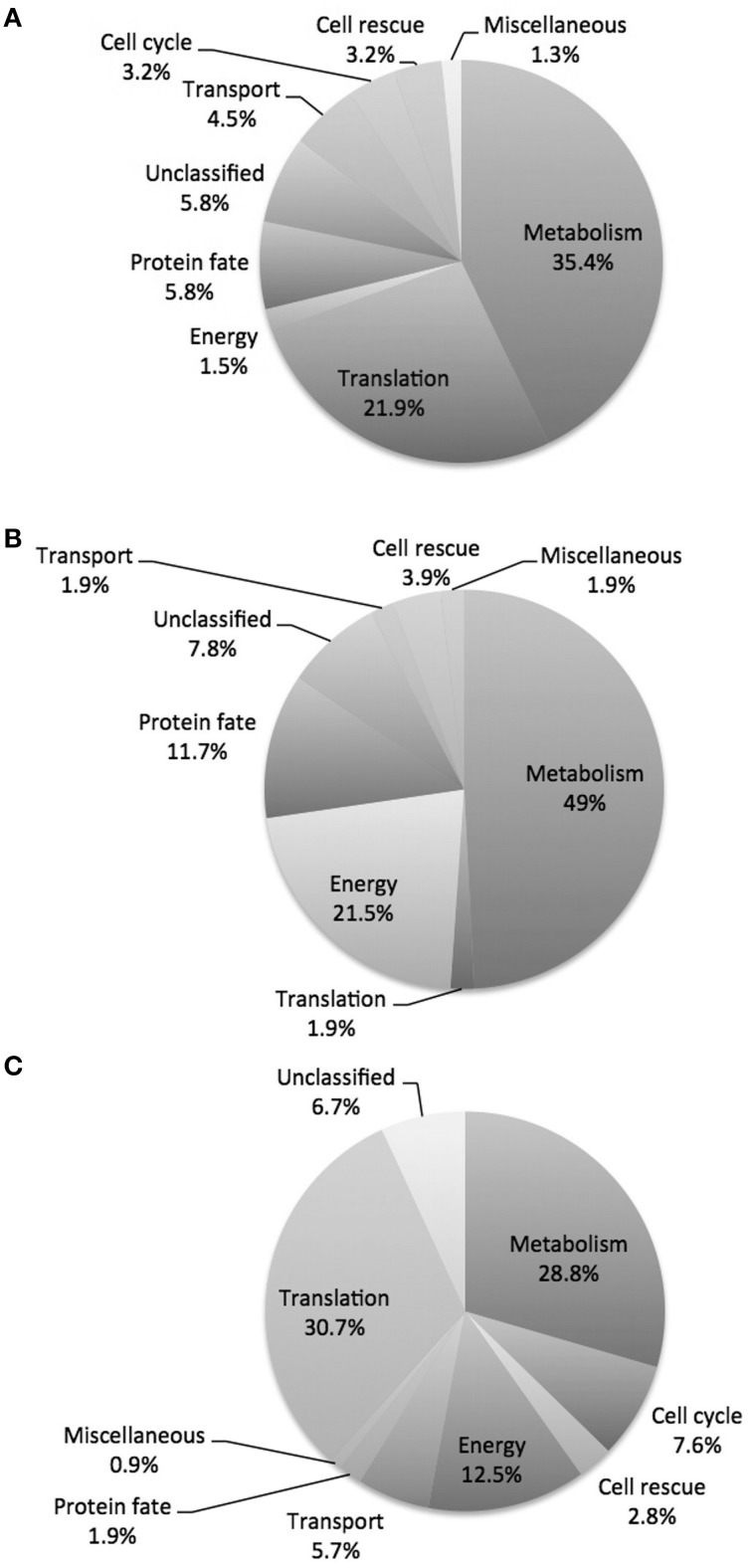
**Diagram depicting the breakdown of** ***P. lutzii*** **proteins. (A)** Proteins differentially expressed in the absence and presence of argentilactone; **(B)** More abundant proteins in the presence of argentilactone; **(C)** Less abundant proteins in the absence of argentilactone.

The proteomic analysis, including all regulated proteins, showed proteins associated with metabolism 35.4%, translation 21.9%, protein fate 5.8%, unclassified 5.8%, transport 4.5%, cell cycle 3.2%, cell rescue 3.2%, energy 1.5% and miscellaneous 1.3% (Figure 4A; Tables 1, 2). The proteome analysis that included up-regulation and proteins exclusive to the presence of argentilactone showed proteins associated with metabolism 49%, energy 21.5%, protein fate 11.7%, unclassified 7.8%, cell rescue 3.9%, transport 1.9%, translation 1.9% and miscellaneous 1.9% (Figure 4B; Table 1). The proteome analysis that included down-regulation and proteins exclusive to the control condition showed proteins associated with translation 30.7%, metabolism 28.8%, energy 12.5%, cell cycle 7.6%, unclassified 6.7%, transport 5.7%, cell rescue 2.8%, protein fate 1.9% and miscellaneous 0.9% (Figure 4C; Table 2).

The proteins involved in cell rescue, defense, and virulence confer protection to the cell and assure survival upon various stresses. Molecular chaperones are very conserved and has the function related to maintenance of conformational equilibrium of proteins (Hartl, 1996). In this study, as could be expected, were identified stress-related proteins regulated in the presence of argentilactone (Tables 1, 2). In addition to the heat shock proteins, proteasome component C5 and sulfur metabolite repression control protein C were exclusive to *P. lutzii* exposed to argentilactone. This result could indicate the involvement of these proteins in protecting the fungus from the stress generated by argentilactone.

**Figure 5:**
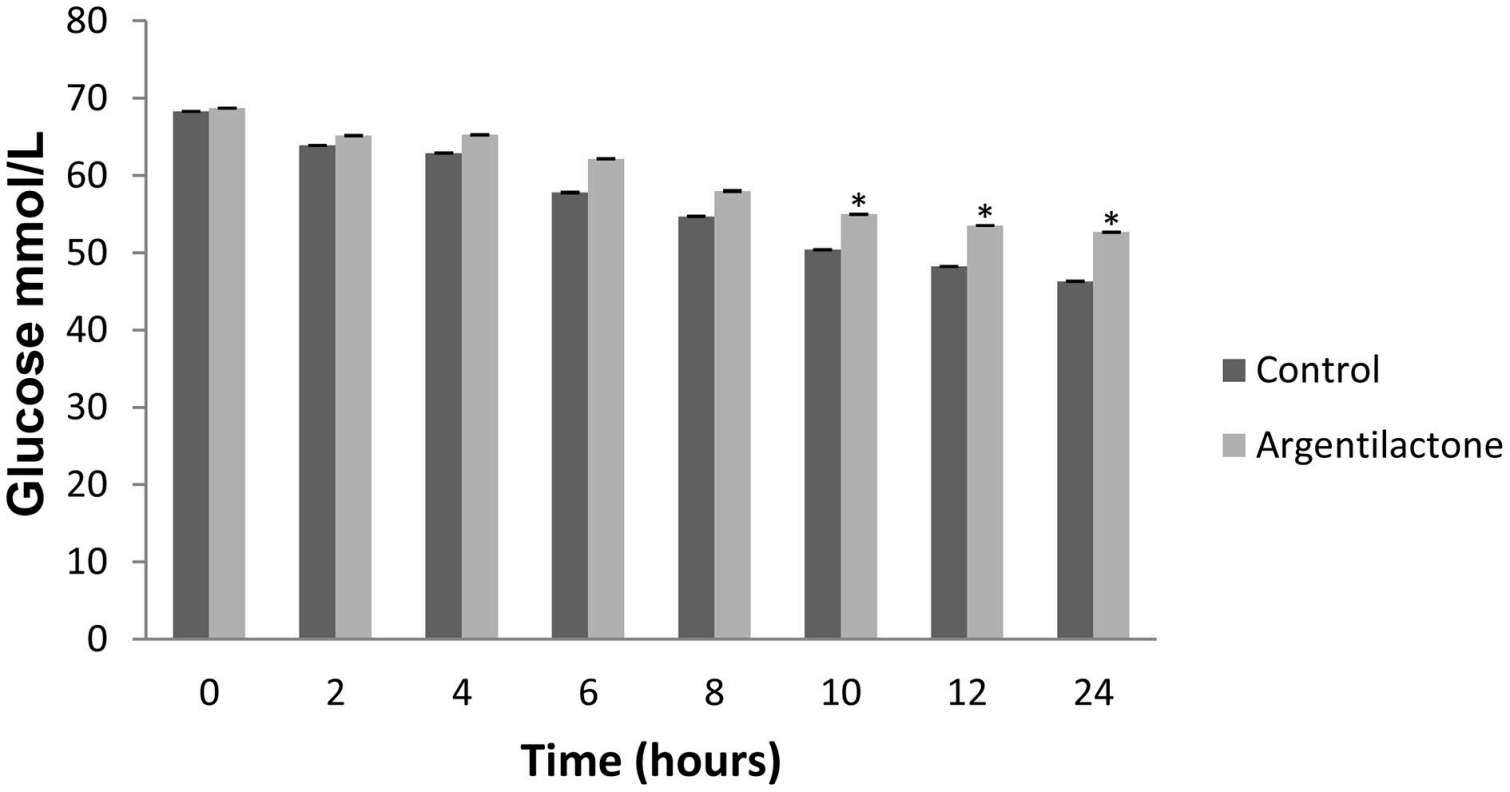
**Glucose quantification**. The level of glucose was quantified by enzymatic kit after 0, 2, 4, 6, 8, 10, 12 and 24 h. The control was performed with cells in the absence of argentilactone. The Student's *t*-test was used for statistical comparisons, and the observed differences were statistically significant (^*^p ≤ 0.05).

Our proteomic analyses indicate a global reorganization of *P. lutzii* carbohydrate metabolism during the exposure to argentilactone. One change detected here is the decrease of several enzymes of glycolytic pathway such as enolase, phosphoglucomutase, phosphoglycerate kinase, pyruvate kinase, and those exclusive to the absence of argentilactone as glucokinase and phosphofructokinase (Table 2). The down-regulation of succinate dehydrogenase, two malate dehydrogenases, and isocitrate dehydrogenase subunits 1 and 2 (Table 2), shows that Krebs cycle is not completely functioning in *P. lutzii*. In the presence of argentilactone, *P. lutzii* decreased the glucose consume (Figure 5), suggesting that glycolysis is partially blocked. In addition, the gluconeogenesis is also not completely functioning, as phosphoenolpyruvate carboxykinase is less abundant (Table 2). Phosphoenolpyruvate carboxykinase plays an importantl role in the pathogenesis of tuberculosis, sinceit is essential for *Mycobacterium tuberculosis* during mouse infection. *M. tuberculosis* utilizes primarily gluconeogenic substrates for *in vivo* persistence, suggesting that this enzyme represents a target for treatments (Marrero et al., 2010).

The glyoxylate cycle is not completely functioning in the presence of argentilactone as the enzyme isocitrate lyase is less abundant (Table 2). This finding is consistent with our previous results showing that the *P. lutzii* isocitrate lyase recombinant and native forms were inhibited in the presence of argentilactone (Prado et al., 2014). On the other hand, malate synthase is more abundant. Under the absence of six-carbon elements, the glyoxylate cycle is induced (Fernandez et al., 1993). The glyoxylate pathway is important in the generations of C4 dicarboxylic acids from acetyl-CoA units, bypassing the decarboxylation steps in the TCA cycle. The cycle is important to fungal pathogenesis. For example, many of the genes highly induced in phagocytized *C. albicans* were members of the glyoxylate cycle (Lorenz and Fink, 2001; Lorenz et al., 2004). The *C. albicans* isocitrate lyase gene is essential for gluconeogenic carbon source utilization and starvation rather than a marker for lipid metabolism (Brock, 2009; Otzen et al., 2013).

The methylcitrate cycle is an alternative route of carbon through pyruvate production (Bramer et al., 2002) and an important pathway for propionyl-CoA metabolism is the methylcitrate pathway. The 2-methylcitrate dehydratase that participates in the methylcitrate cycle is more abundant (Table 1). In addition, methylmalonate-semialdehyde dehydrogenase that produces propionyl-CoA seems to lead to the production of pyruvate (Table 1). Pyruvate produces acetaldehyde from the action of pyruvate decarboxylase that is more abundant in the presence of argentilactone (Table 1). Up-regulation of *o*-acetylhomoserine (thiol)-lyase leads to the production of L-methionine and acetate. Acetate is converted to acetoacetyl-CoA by the action of acetyl-CoA acetyltransferase, which was only detected during the treatment with argentilactone (Table 1).

**Figure 6:**
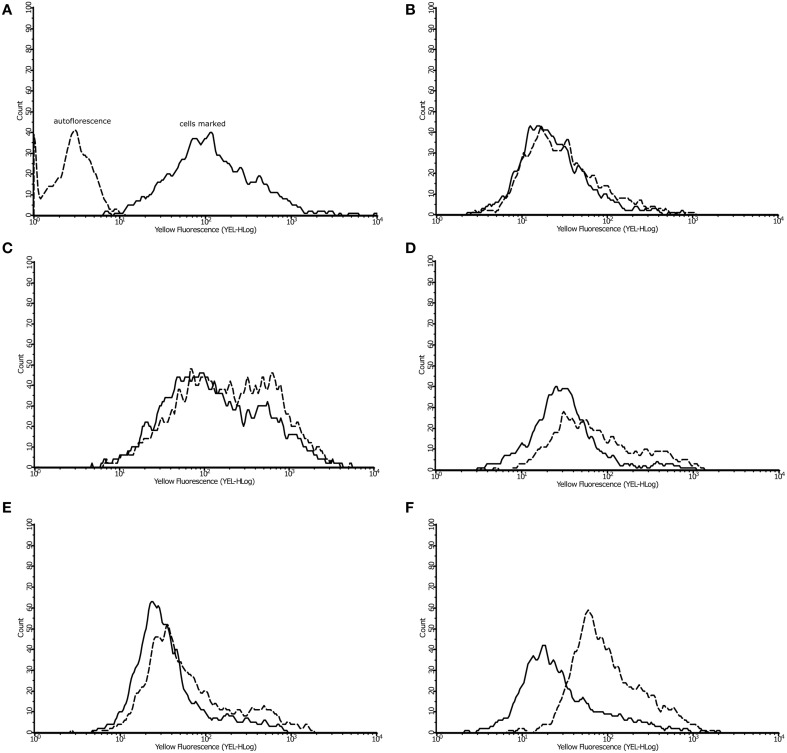
**Effect of argentilactone on intracellular lipid content of** ***P. lutzzi***. The presence of lipids was determined by flow cytometry. Cells was stained with dye Nile Red **(A)**. The analysis of yeast cells in presence and absence of argentilactone for **(B)** 0 h, **(C)** 6 h, **(D)** 10 h, **(E)** 12 h, and **(F)** 24 h was performed. Line histograms represent the cells treated with argentilactone and dotted histograms represent control cells without treatment.

The β-oxidation is a pathway for the utilization of fatty acids (Poirier et al., 2006) in which the 3-ketoacyl-CoA thiolases enzymes are so important (Otzen et al., 2013). The enzymes 3-ketoacyl-CoA thiolase, which was only detected in *P. lutzii* exposed to argentilactone, and enoyl-CoA hydratase from β-oxidation were also more abundant (Table 1). The lipids content from *P. lutzzi* was decreased in the presence of argentilactone mainly after 24 h (Figure 6) reinforcing the importance of the β-oxidation and methylcitrate cycle for *P. lutzzi* responding to argentilactone.

Glyoxylate is not produced from isocitrate because isocitrate lyase is less abundant in the presence of argentilactone. The high production of succinate is indicated by up-regulation of fumarylacetoacetase, which uses 4-fumarylacetoacetate to produce fumarate, and then fumarate reductase uses fumarate to produce succinate (Table 1).

**Figure 7:**
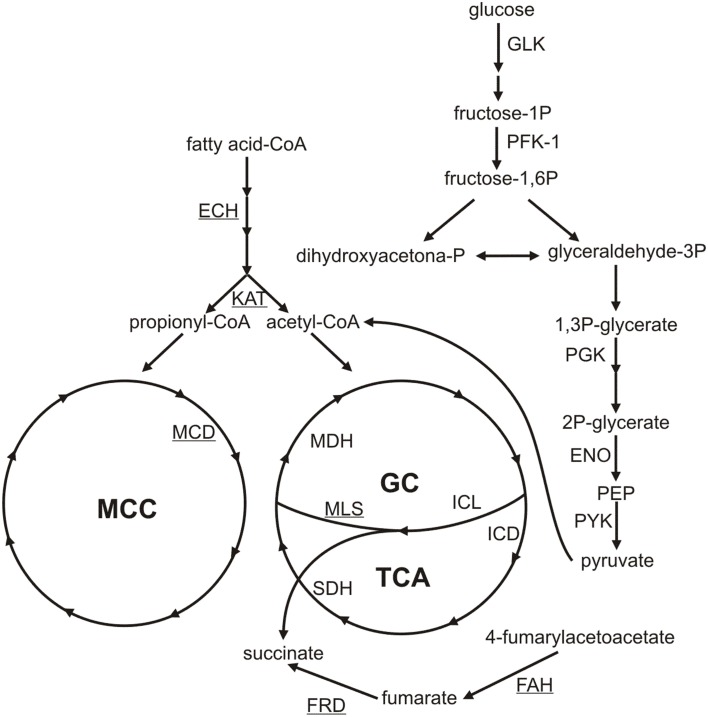
**Metabolic changes of** ***P. lutzii*** **yeast cells exposed to argentilactone**. The less abundant proteins during treatment are not highlighted. The more abundant proteins are underlined. GC, glyoxylate cycle; TCA, tricarboxylic acid cycle; MCC, methylcitrate cycle; GLK, glucokinase; PFK-1, phosphofructokinase-1; PGK, phosphoglycerate kinase; ENO, enolase; PYK, pyruvate kinase; ICL, isocitrate lyase; MLS, malate synthase; MDH, malate dehydrogenase; FAH, fumarylacetoacetase; FRD, fumarate reductase; ECH: enoyl-CoA-hydratase; KAT, acetyl-CoA acetyltransferase; SDH, succinate dehydrogenase; IDH, isocitrate dehydrogenase; MCD, methylcitrate dehydrogenase.

It is important to mention that argentilactone weakened the protein synthesis of *P. lutzii*. Translation was the functional category most affected with 33 less abundant proteins. In general, we could observe that energy-producing pathways, such as glycolysis, gluconeogenesis, and TCA, were less abundant in the presence of argentilactone. An overview of the metabolic changes of *P. lutzii* in presence of the compound is shown in Figure 7.

### Validation of nanoUPLC-MS^E^ data

The innate immune cells like resident macrophages and dendritic cells are the first barriers of defense system that interact with *Paracoccidioides* spp. cells (Calich et al., 2008). It is known that the phagosome is poor in nutrients and was reported to not are a good environment as evidenced by the little quantities of glucose, other sugars, and amino acids (Lorenz et al., 2004; Fan et al., 2005; Tavares et al., 2007; Cooney and Klein, 2008; Silva et al., 2008).

**Figure 8:**
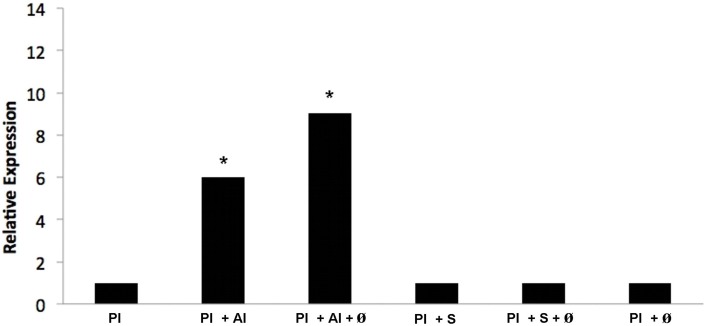
**Quantification of the mRNA expression of the methylcitrate dehydrogenase gene of** ***P. lutzii*** **infecting macrophage during exposure to argentilactone and sulfamethoxazole by quantitative qRT-PCR**. (1) *P. lutzii* (Pl); (2) *P. lutzii* (Pl) + argentilactone (Al); (3) *P. lutzii* (Pl) + argentilactone (Al) + ø; (4) *P. lutzii* (Pl) + sulfamethoxazole (S); (5) *P. lutzii* (Pl) + sulfamethoxazole (S) + ø; (6) *P. lutzii*(Pl) + ø. Data were normalized to the tubulin transcript. Data were analyzed by a One-Way ANOVA and a Tukey's multiple comparison post-test. ^*^p ≤ 0.05.

Methylcitrate dehydrogenase is an important enzyme of the methylcitrate cycle. Thus, aiming to verify whether the transcript is regulated *in vivo* when *P. lutzii* is exposed to argentilactone, the compound was added to the medium during J744 A.1 macrophage infection. The relative expression analysis of transcripts encoding methylcitrate dehydrogenase was performed using qRT-PCR. Figure 8 shows that genes encoding methylcitrate dehydrogenase were induced, corroborating the observations from proteomic data. This finding indicates that the methylcitrate cycle composes the response of yeast cells during macrophage infection and not only *in vitro*.

## Conclusions

The global characterization of the proteomic profile of *P. lutzii* responding to argentilactone enabled the visualization of the metabolic adaptation of the fungus to drug exposure. Important metabolic pathways were regulated, explaining the strong action of the compound on fungus growth and viability. In this study, alternative metabolic pathways adopted by the fungi were elucidated and helped to elucidate the course of action of the compound studied.

## Funding

This work performed at Universidade Federal de Goiás was supported by MCTI/CNPq (Ministério da Ciência e Tecnologia/Conselho Nacional de Desenvolvimento Científico e Tecnológico), FNDCT (Fundo Nacional de Desenvolvimento Científico e Tecnológico), FAPEG (Fundação de Amparo à Pesquisa do Estado de Goiás), CAPES (Coordenação de Aperfeiçoamento de Pessoal de Nível Superior), FINEP (Financiadora de Estudos e Projetos), and INCT-IF (Instituto Nacional de Ciência e Tecnologia para Inovação Farmacêutica). Additionally, FSA and BRSN were supported by fellowship from CAPES.

### Conflict of interest statement

The authors declare that the research was conducted in the absence of any commercial or financial relationships that could be construed as a potential conflict of interest.
